# Case Report: Simultaneous presence of *Sarcoptes scabiei* and fungal elements in scrapings from a patient with bullous pemphigoid

**DOI:** 10.3389/fmed.2026.1773134

**Published:** 2026-03-09

**Authors:** Yifeng Ye, Wenwen Lv, Li Chai, Qiuping Li, Zehu Liu, Xiujiao Xia

**Affiliations:** 1 Department of Clinical Laboratory, Hangzhou Third People’s Hospital, Hangzhou Third Hospital Affiliated to Zhejiang Chinese Medical University, Hangzhou, China; 2 Department of Dermatology, Hangzhou Third People’s Hospital, Hangzhou Third Hospital Affiliated to Zhejiang Chinese Medical University, Hangzhou, China

**Keywords:** bullous pemphigoid, *Candida albicans*, case report, crusted scabies, glucocorticoid

## Abstract

Glucocorticoids exert their therapeutic effects by broadly suppressing the activity of immune cells, thereby increasing the risk of infection. Although this risk is dose-dependent, a clinically meaningful increase in infection risk persists even at daily doses lower than 5 mg of prednisone equivalent. We herein report the first documented case of a patient who developed concurrent crusted scabies and *Candida albicans* infection following glucocorticoid therapy for bullous pemphigoid.

## 1 Introduction

Bullous pemphigoid (BP) is the most frequent autoimmune blistering disorder (1). BP is characterized by the presence of autoantibodies against basement membrane zone (BMZ) autoantigens, including BP180 (180 kDa, also known as BPAG2) and BP230 (230 kDa, also known as BPAG1). Both antigens are important components of hemidesmosomes responsible for maintaining the adhesion function between epidermis and dermis (2). Glucocorticoids represent the standard therapy for reducing inflammation and immune activation across a wide spectrum of diseases. Their clinical applications include the management of asthma, allergic disorders, rheumatoid arthritis, collagen vascular diseases, dermatological conditions, inflammatory bowel disease, other systemic autoimmune disorders, and ocular inflammatory diseases. While these agents possess potent anti-inflammatory and immunosuppressive actions, they are also associated with potentially undesirable side effects (3, 4). We report a rare case of concurrent crusted scabies and cutaneous *Candida albicans* infection after glucocorticoid treatment of BP.

## 2 Case description

**Figure 1:**
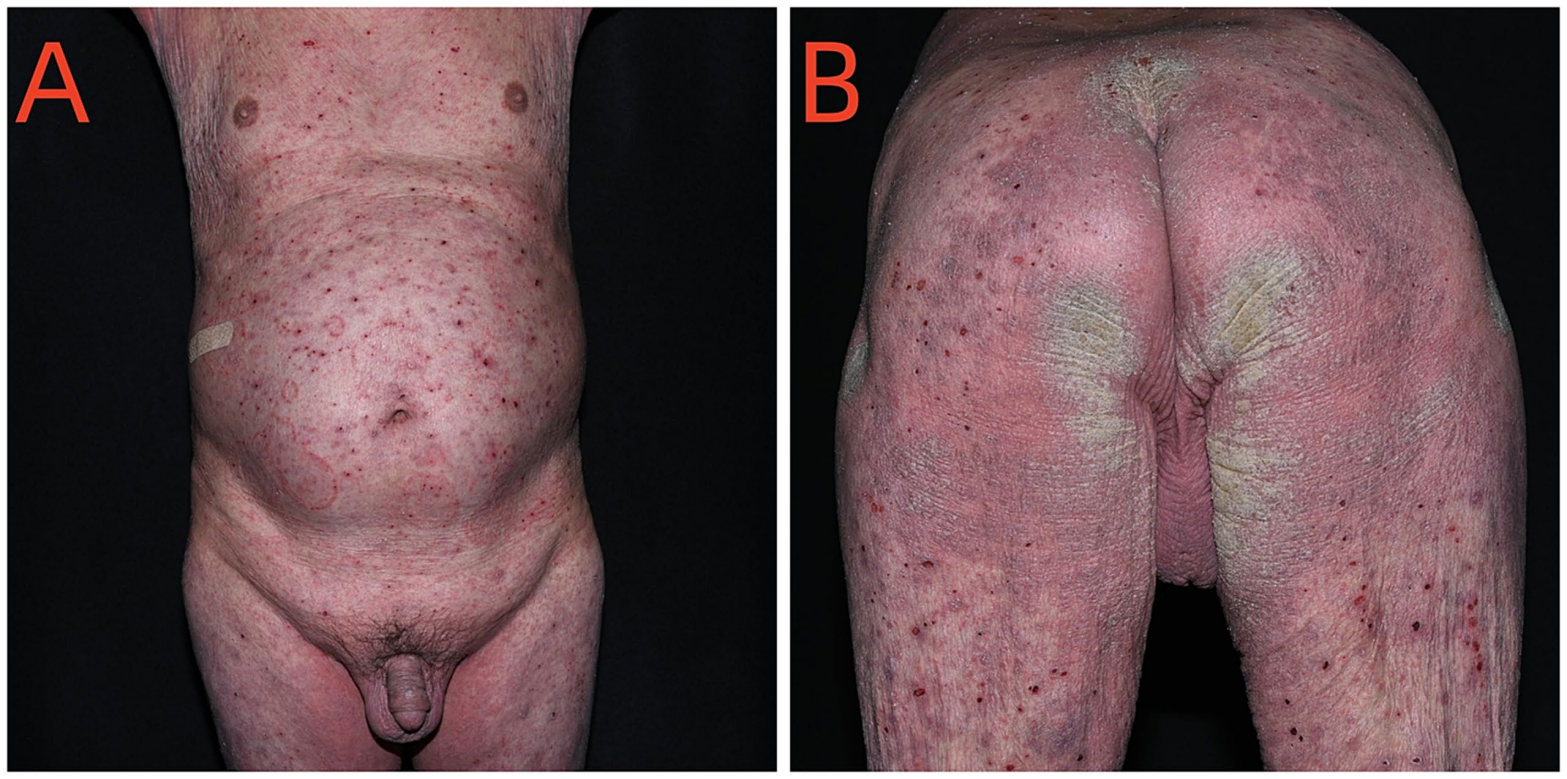
Initial clinical manifestations after 5 months of systemic steroid therapy. **(A)** Diffuse papules rash and erythematous plaques with well-defined borders located on the abdomen; **(B)** Hyperkeratotic and crusted plaques over bilateral buttocks.

**Figure 2:**
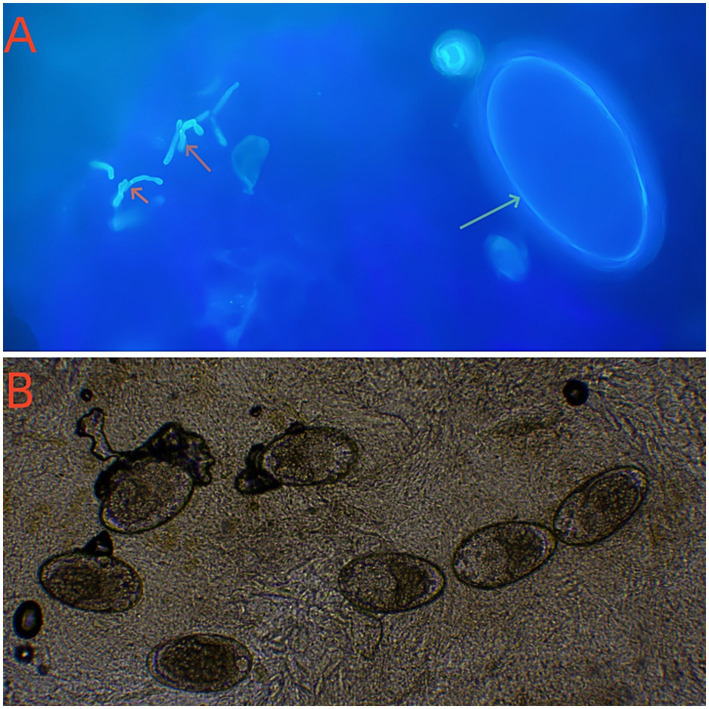
Direct microscopic examination of skin scrapings revealed the presence of both *Sarcoptes scabiei* mites and fungal elements on the same smear. **(A)** Optical microscopy of the scrapings from plaques of the patient’s buttock showing egg of scabies mite (green arrow) and pseudohyphae (red arrow)in the same visual field (Calcofluor white × 400); **(B)** Numerous eggs of scabies mite (10% potassium hydroxide solution × 100).

**Figure 3:**
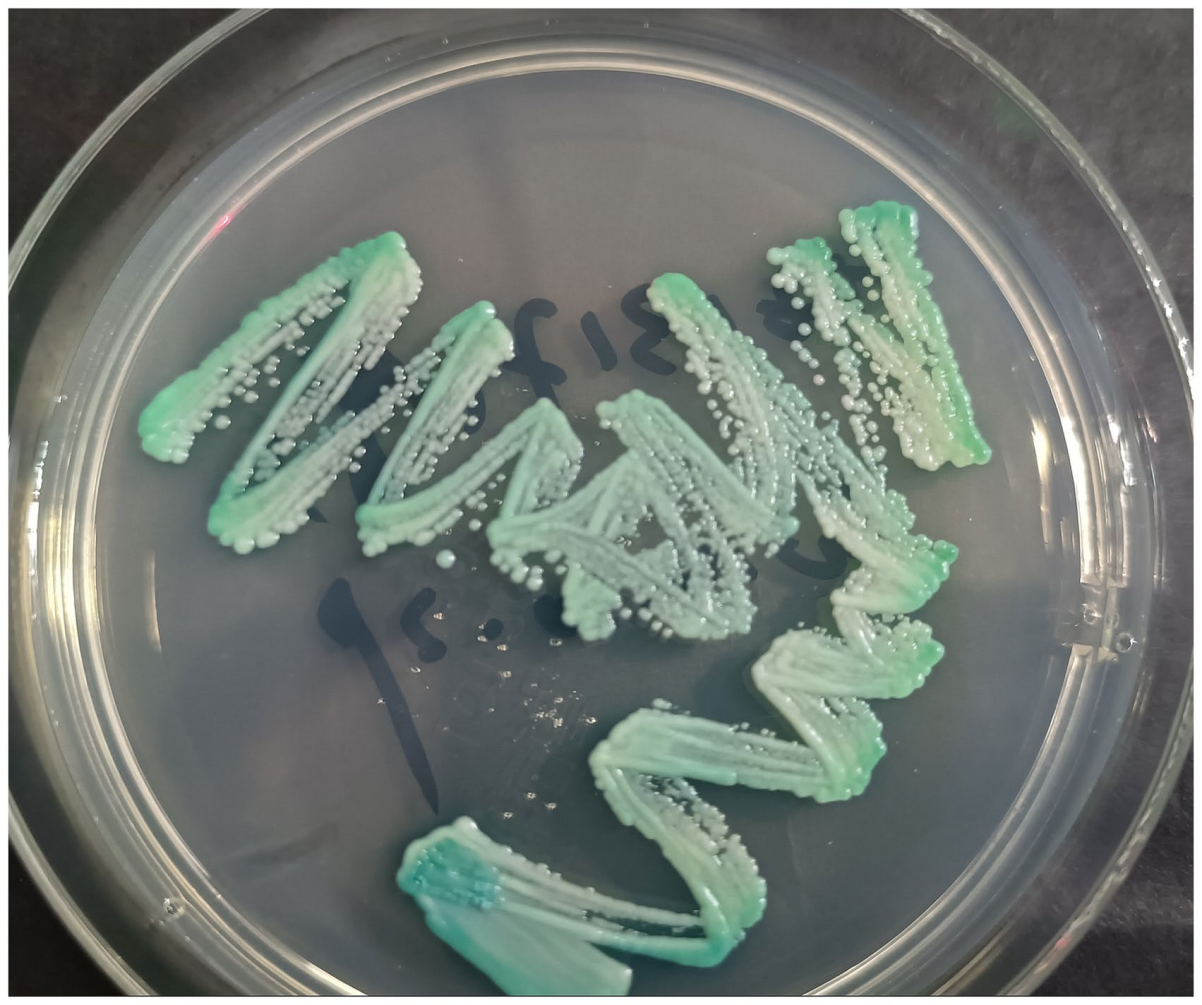
Green colonies of *C. albicans* on the CHROMagar Candida plate.

On October 21, 2024, a 70-year-old male weighing 62 kg presented to the Dermatology Department with a chief complaint of pruritic eruptions occurring throughout the body. Six months prior, he had developed blisters and erythema on the trunk and limbs and was presumptively diagnosed with bullous pemphigoid at an external hospital. He was treated with oral methylprednisolone (24 mg/day) for 5 months, which resulted in improvement of the skin lesions. However, he recently developed pruritic eruptions all over the body. Dermatological examination revealed diffuse papular rashes and well-demarcated erythematous plaques on the chest, abdomen, bilateral palms, finger web spaces, axillae, lower limbs, and inguinal region. Multiple hyperkeratotic and crusted plaques were also observed on both buttocks (Figures 1A,B). Investigation of humoral immunity showed an increased serum IgE (1782 IU/ mL, normal 0–100). Routine laboratory abnormalities included a leukocyte count of 14.0 × 10^9^/L, a neutrophil count of 11.37 × 10^9^/L, and a Helper T cells (CD4+) count of 391 cells/mm^3^ (normal range 410–1,440 cells/mm^3^). The remaining laboratory findings, including HIV testing, were essentially within normal limits. Direct microscopic examination of skin scrapings from his buttocks, abdomen and hands revealed numerous mites and eggs, as well as fungal pseudohyphae (Figures 2A,B). Fungal cultures revealed colonies of *C. albicans* (Figure 3). Based on these findings, a diagnosis of crusted scabies with cutaneous *C. albicans* infection was established. Corticosteroid therapy was discontinued, and treatment was initiated with a topical scabicide (topical compound sulfur cream) and an antifungal agent (oral itraconazole 200 mg/day). After 10 days of treatment, the skin lesions showed significant improvement.

**Figure 4:**
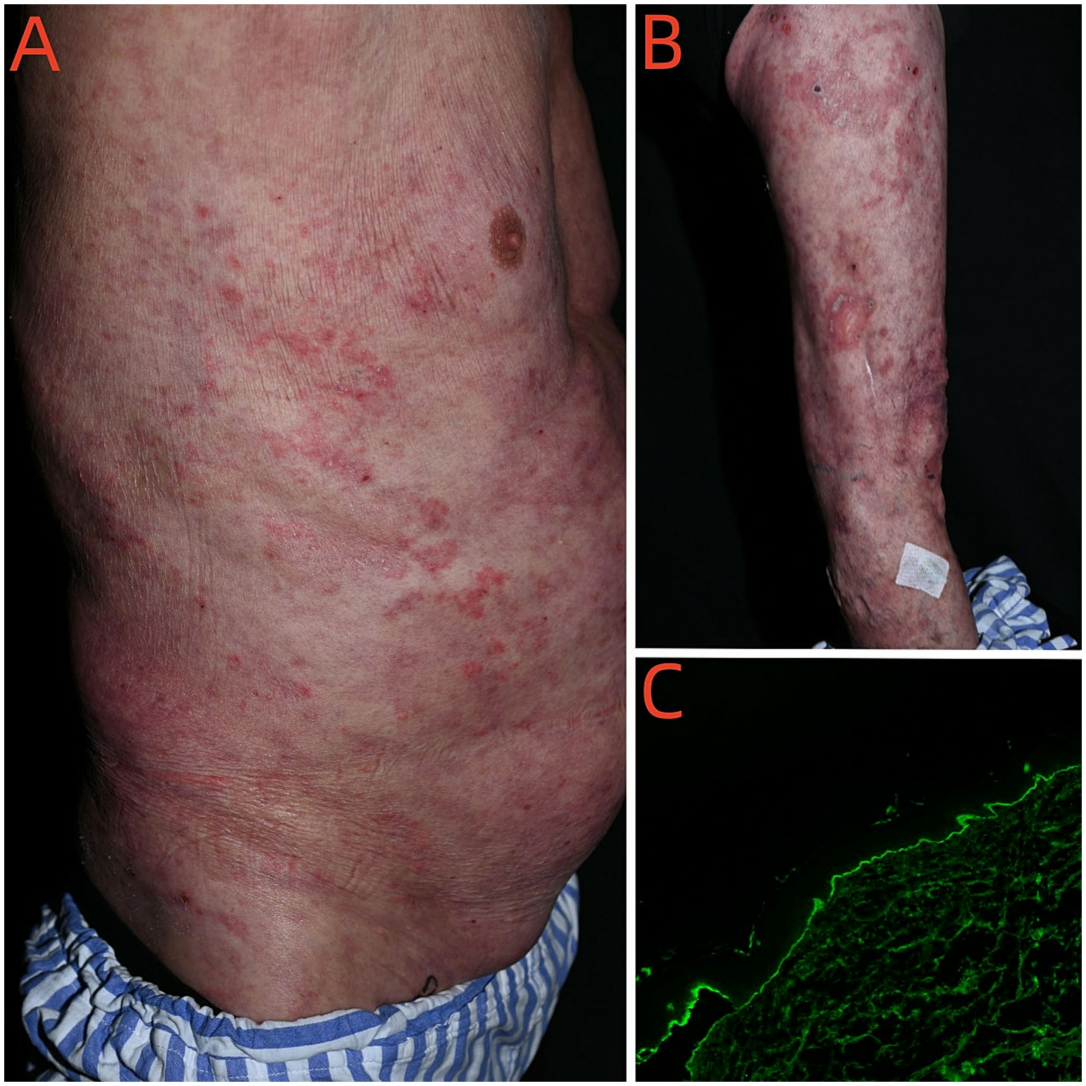
New-onset skin lesions after cure of crusted scabies **(A,B)**. Linear IgG deposits in the basement membrane zone (DIF × 100). **(C)** Linear IgG deposits in the basement membrane zone (DIF × 100).

On December 16, 2024, the patient was admitted to the hospital due to the recurrence of widespread erythema and blisters. Physical examination revealed numerous erythematous macules and patches and dark erythematous patches of varying sizes on the skin of the trunk and limbs. Erosions were present on some of the erythematous bases. A single tense bulla with clear fluid and a thick wall was observed on the right thigh (Figures 4A,B); Nikolsky’s sign was negative. Erosions and crusted areas were partially visible. A single erosion was noted on the oral mucosa. The scrotum showed no blisters or erosions. No nodules, cysts, petechiae, ecchymoses, ulcers, or necrotic areas were present. The Bullous Pemphigoid Disease Area Index (BPDAI) score was 30. Skin biopsies from the back were performed. Histopathological examination revealed hyperkeratosis with focal serocellular crusting containing neutrophils within the stratum corneum. Mild acanthosis was present, along with focal vacuolar degeneration of the basal cell layer. Focal subepidermal cleft formation was observed. The superficial dermis showed a perivascular infiltrate of moderate to sparse lymphocytes, neutrophils, and a few melanophages, accompanied by scattered extravasated red blood cells. Direct immunofluorescence (DIF) showed linear IgG and focal C3 deposits in the basement membrane zone (Figure 4C). Serum testing for five pemphigus/pemphigoid autoantibodies revealed the following: BP230 antibody 2 RU/mL, anti-basement membrane zone antibody negative, desmoglein 1 (Dsg1) antibody 2 RU/mL, desmoglein 3 (Dsg3) antibody 3 RU/mL, and BP180 antibody 63 RU/mL (normal reference range: <20 RU/mL). The patient was administered oral methylprednisolone tablets at a dose of 40 mg/day for anti-inflammatory therapy. The corticosteroid dose was tapered to 24 mg/day on December 21. By December 24, the skin lesions had regressed compared to admission, pruritus was alleviated, and no significant new lesions were observed. The patient was subsequently discharged and continued on oral methylprednisolone tablets at a maintenance dose of 24 mg/day. The patient was subsequently lost to follow-up.

## 3 Discussion

The primary goals of treating BP are to suppress the formation of new lesions, facilitate cutaneous healing, and control pruritus. Despite the emergence of steroid-sparing agents, systemic corticosteroids remain a first-line therapy in several treatment guidelines, particularly for managing severe or widespread disease (5–7). Glucocorticoids exert their therapeutic effects through broad suppression of immune cell activity. However, prolonged high-dose regimens can induce a state of multifaceted immune dysfunction. This is characterized by leukocyte apoptosis, impaired immunoregulation, and a diminished capacity to respond to pathogens (8), consequently predisposing patients to a spectrum of opportunistic infections, including those of viral, bacterial, mycotic, and parasitic origin (9). While this infectious risk is dose-dependent, a clinically meaningful increase in susceptibility persists even at daily doses below 5 mg of prednisone-equivalent. Observational studies consistently corroborate these associations, demonstrating a correlation between infection risk and both the magnitude of the glucocorticoid dose and the duration of treatment (10). Given this risk profile, the total daily dosage of oral steroids is critically important and is typically determined based on the patient’s body weight (11, 12). To mitigate the potential for increased adverse events and mortality, Morel et al. recommend avoiding the use of prednisolone or prednisone at doses exceeding 0.75 mg/kg/day (13).

Crusted scabies (Norwegian scabies) represents an extreme form of *Sarcoptes scabiei* infestation marked by hyperkeratotic lesions containing thousands of mites. This severe variant predominantly affects immunocompromised populations, particularly those undergoing steroid therapy, HIV/HTLV-1 patients, transplant recipients, and individuals with significant physical or cognitive impairments (14). We herein describe the first known case of a patient who developed concurrent crusted scabies and *C. albicans* infection following glucocorticoid treatment for BP. The presented case demonstrates notable clinical complexity: well-demarcated erythematous plaques on the trunk contrasted sharply with hyperkeratotic lesions on the gluteal region, with microscopic examination revealing concurrent mite infestation and fungal colonization. *Candida* spp., particularly *C. albicans*, exist as commensal organisms but can transition to pathogenic states under conditions of immunosuppression or microbiota disruption from broad-spectrum antimicrobials (15). The case underscores the critical need for vigilant infection monitoring when implementing potent immunosuppressive treatments.

Given that bullous pemphigoid (BP) may present with polymorphic clinical features, including both non-bullous manifestations and typical blisters, a broad range of differential diagnoses should be considered. These include: pemphigus foliaceus, pemphigus herpetiformis, linear IgA bullous dermatosis, epidermolysis bullosa acquisita, bullous lupus erythematosus, eczema, urticaria, prurigo, impetigo, erythema multiforme, Sweet syndrome, toxic epidermal necrolysis, and autotoxic pruritus (16). Bullous scabies is an atypical manifestation of scabies. It may appear concurrently with typical scabies lesions or develop subsequent to them (17, 18). Bullous scabies is characterized by the presence of bullae in the same anatomical regions typically affected by the classic form of the infestation. These fluid-filled blisters may be flaccid or tense, often exceeding 5 mm in diameter, and may or may not be associated with pruritus (19). The manifestations of atypical scabies are varied; for instance, Izmailovich et al. reported a case of vesicular toxicodermia caused by *S. scabiei* (20), which is worthy of attention.

Additionally, scabies is a cutaneous infectious inflammatory disease. Infestation with Sarcoptes scabiei induces host immune responses, and several alterations in cytokine profiles have been reported in previous studies (21, 22). Immune-mediated inflammatory processes have been implicated in both scabies infection and autoimmune diseases in the existing literature and may contribute to their potential associations (23). In our case, the recurrence of bullous pemphigoid (BP) shortly after the control of scabies raises the question of whether scabies may trigger autoimmune responses—possibly through the exposure of cutaneous antigens following damage to the epidermis and basement membrane caused by the burrowing of Sarcoptes mites—an intriguing hypothesis that warrants further investigation.

Recent studies have further evaluated the role of adjuvant therapies aimed at reducing the cumulative dose of systemic steroids or even replacing steroid-based regimens as the standard of care (24). For example, doxycycline has been employed as an adjuvant therapy in BP owing to its anti-inflammatory properties, which do not induce significant immunosuppression. Its mechanism involves inhibition of matrix metalloproteinases and neutrophilic activation triggered by immune complex formation between IgG autoantibodies and BP antigens. This process helps prevent disruption of the dermal-epidermal junction and may contribute to improved disease control (25). Moreover, biologics with more specific targets relevant to the pathogenesis of BP have also emerged as alternative therapeutic options (16).

## Data Availability

The raw data supporting the conclusions of this article will be made available by the authors, without undue reservation.
